# Clinical Impact of Baseline ctDNA *RAS*/*BRAF* Mutations on Conversion Surgery and Outcomes in First-Line Anti-EGFR Therapy for Advanced Colorectal Cancer

**DOI:** 10.3390/cancers18111688

**Published:** 2026-05-22

**Authors:** Takeshi Yamada, Takeshi Nagasaka, Nobuhisa Matsuhashi, Takao Takahashi, Keiji Hirata, Yuki Nakamura, Kiichi Sugimoto, Keiji Koda, Kazuhiro Hiramatsu, Hiroshi Matsuoka, Hidekazu Kuramochi, Akihisa Matsuda, Hideyuki Ishida, Kozo Kataoka, Hajime Yokomizo, Yoshinori Kagawa, Mitsukuni Suenaga, Hiroshi Yoshida

**Affiliations:** 1 Department of Gastroenterological Surgery, Nippon Medical School, 1-1-5 Sendagi, Bunkyo-ku, Tokyo 113-8603, Japan; a-matsu@nms.ac.jp (A.M.);; 2 Department of Advanced Oncology, Kawasaki Medical School, 577 Matsushima, Kurashiki 701-0192, Japan; takeshin@med.kawasaki-m.ac.jp; 3 Department of Gastroenterological Surgery and Pediatric Surgery, Gifu University Graduate School of Medicine, 1-1 Yanagido, Gifu 501-1194, Japan; matsuhashi.nobuhisa.k6@f.gifu-u.ac.jp; 4 Department of Surgery, Seino Kosei Hospital, Gifu Seino Medical Center, 293-1 Shimoiso, Ohno-cho, Gifu 501-0532, Japan; takaota@gfkosei.or.jp; 5 Department of Surgery 1, School of Medicine, University of Occupational and Environmental Health, 1-1 Iseigaoka, Yahatanishi-ku, Kitakyushu 807-8555, Japan; hirata@med.uoeh-u.ac.jp; 6 Second Department of Surgery, Wakayama Medical University, 811-1 Kimiidera, Wakayama 641-8509, Japan; y-nakamu@wakayama-med.ac.jp; 7 Department of Coloproctological Surgery, Juntendo University Faculty of Medicine, 2-1-1 Hongo, Bunkyo-ku, Tokyo 113-8421, Japan; 8 Department of Surgery, Faculty of Medicine, Teikyo University, 2-11-1 Kaga, Itabashi-ku, Tokyo 173-8605, Japan; k-koda@med.teikyo-u.ac.jp; 9 Department of General Surgery, Toyohashi Municipal Hospital, 50 Aza Hachiken-nishi, Aotake-cho, Toyohashi 441-8570, Japan; 10 Department of Gastrointestinal Surgery, Fujita Health University, 1-98 Dengakugakubo, Kutsukake-cho, Toyoake 470-1192, Japan; 11 Department of Medical Oncology, NTT East Japan Kanto Hospital, 5-9-22 Higashi-Gotanda, Shinagawa-ku, Tokyo 141-8625, Japan; 12 Department of Digestive Tract and General Surgery, Saitama Medical University General Medical Center, 1981 Kamoda, Kawagoe 350-8550, Japan; 13 Division of Lower GI Surgery, Department of Gastroenterological Surgery, Hyogo Medical University, 1-1 Mukogawa-cho, Nishinomiya 663-8501, Japan; 14 Department of Surgery, Tokyo Women’s Medical University Adachi Medical Center, 4-33-1 Kohoku, Adachi-ku, Tokyo 123-8558, Japan; 15 Department of Gastroenterological Surgery, Osaka International Cancer Institute, 3-1-69 Otemae, Chuo-ku, Osaka 541-8567, Japan; 16 Department of Clinical Oncology, Institute of Science Tokyo, 2-12-1 Ookayama, Meguro-ku, Tokyo 152-8550, Japan

**Keywords:** metastatic colorectal cancer, ctDNA, liquid biopsy, RAS, BRAF, EGFR blockade, conversion surgery

## Abstract

Background: Baseline circulating tumor DNA (ctDNA) testing may detect minor resistant subclones in patients whose tumor tissue is classified as *RAS*/*BRAF* wild-type, but its clinical significance during first-line anti-EGFR therapy remains uncertain. Methods: We prospectively enrolled 98 patients with tissue-confirmed *RAS*/*BRAF* wild-type stage IV colorectal cancer treated with first-line anti-EGFR-based chemotherapy at multiple centers. Pretreatment plasma was analyzed by droplet digital PCR for *KRAS*, *NRAS*, and *BRAF* mutations; *PIK3CA* was assessed exploratorily. Results: Baseline ctDNA *RAS*/*BRAF* mutations were detected in 16 patients (16.3%; 95% CI, 10.3–24.9%), and any mutation including *PIK3CA* was detected in 20 (20.4%). Among 88 patients with measurable disease, objective response rates were similar in concordant and discordant groups (84.7% vs. 81.3%), as were conversion surgery rates (48.6% vs. 56.3%). Median progression-free survival was 14.0 months in both groups. Median overall survival was 44.5 and 33.8 months, respectively, without a statistically significant difference (log-rank *p* = 0.20). Conclusions: Restricted baseline ddPCR targeting *RAS*/*BRAF* did not reliably identify patients who would fail to achieve early tumor shrinkage or conversion surgery, and these results do not support withholding first-line anti-EGFR-based induction therapy solely on the basis of minor baseline ctDNA *RAS*/*BRAF* mutations detected by limited targeted testing. Given the limited number of discordant cases, however, a clinically meaningful prognostic role of baseline ctDNA discordance cannot be excluded, and findings from restricted hotspot testing should be contextualized within comprehensive NGS-based molecular profiling.

## Simple Summary

Among patients with tissue-confirmed *RAS*/*BRAF* wild-type metastatic colorectal cancer receiving first-line anti-EGFR-based combination therapy, baseline plasma ddPCR detect-ed *RAS*/*BRAF* mutations in 16.3%. Despite this discordance, objective response and con-version surgery remained common, and progression-free survival did not differ signifi-cantly between groups. These findings indicate that restricted baseline hotspot ctDNA testing for *KRAS*, *NRAS*, and *BRAF* is insufficient as a stand-alone exclusion tool for inten-sive anti-EGFR-based induction therapy when early tumor shrinkage and conversion are therapeutic goals.

## 1. Introduction

Epidermal growth factor receptor (EGFR) blockade combined with cytotoxic chemotherapy has substantially improved outcomes in unresectable metastatic colorectal cancer (mCRC), particularly in patients with left-sided *RAS* wild-type disease [1,2,3,4,5]. Over the past decade, the treatment paradigm for mCRC has shifted from purely palliative care to a potentially curative approach for a highly selected subset of patients. Achieving early tumor shrinkage (ETS) and depth of response (DpR) through potent combination chemotherapy is crucial, as it provides the opportunity for conversion surgery [6,7]. Resection of liver or lung metastases following successful induction therapy is currently the most effective strategy to achieve long-term survival and potential cure, offering 5-year survival rates of 30% to 50% [8,9]. Consequently, multidisciplinary team (MDT) management aiming for conversion surgery has become a central therapeutic goal in modern clinical oncology [10].

Nevertheless, a clinically relevant subset of tumors classified as *RAS*/*BRAF* wild-type in tissue derives limited benefit from first-line anti-EGFR therapy. One biologically plausible explanation is spatial and temporal tumor heterogeneity, including the presence of pre-existing resistant subclones that are not captured by routine single-site tissue sampling [11]. However, circulating tumor DNA (ctDNA) detectability is influenced by several biological and technical factors—including tumor burden, anatomical distribution of metastases, organ-specific shedding rates, and assay sensitivity—which can result in both false-negative liquid biopsy results and the detection of subclonal variants unsampled by single-site tissue biopsy [12]. Consistent with the latter, we and others have previously reported that baseline plasma analysis may detect *RAS* or *BRAF* alterations in patients whose tumor tissue was classified as *RAS*/*BRAF* wild-type by tissue-based testing [13,14,15].

However, the increasing sensitivity of liquid biopsy assays raises a profound clinical dilemma: whether the detection of minor *RAS*/*BRAF* mutant subclones should absolutely preclude the use of highly active anti-EGFR antibodies in the first-line setting. While these clones are undoubtedly associated with acquired resistance [16,17], their actual impact on achieving initial tumor shrinkage and subsequent conversion surgery remains poorly defined. Most prior studies assessing the predictive value of ctDNA for EGFR blockade were conducted in later-line settings, used broad next-generation sequencing (NGS) panels, or focused primarily on survival endpoints rather than on surgical conversion [18,19,20].

Recently, the phase III PARADIGM biomarker analysis showed that baseline ctDNA alterations assessed by broad NGS can stratify outcomes of first-line panitumumab-based therapy, supporting negative hyperselection (the strategy of excluding patients from anti-EGFR therapy based on the presence of additional genomic alterations beyond *RAS*/*BRAF*) [20]. In contrast, routine practice often relies on targeted, rapid, and cost-effective ddPCR assays. Whether such restricted *KRAS*/*NRAS*/*BRAF*-focused ddPCR testing is sufficiently informative to exclude patients from potentially curative first-line induction therapy remains unclear. Overtreatment and unnecessary toxicity must be avoided, but so must the inadvertent exclusion of patients from highly effective regimens that may enable surgical salvage.

We therefore conducted a prospective multicenter observational study in patients with tissue-confirmed *RAS*/*BRAF* wild-type stage IV colorectal cancer treated with first-line anti-EGFR-based chemotherapy. The primary aim was to determine the baseline detection rate of ctDNA *RAS*/*BRAF* discordance using focused ddPCR; secondary aims were to explore associations with objective response, conversion surgery, progression-free survival (PFS), and overall survival (OS).

## 2. Materials and Methods

### 2.1. Study Design and Patients

This prospective multicenter observational cohort enrolled adults aged 20–80 years with stage IV colorectal adenocarcinoma whose tumor tissue had been confirmed to be wild-type for both *RAS* and *BRAF*. Eligible patients had an Eastern Cooperative Oncology Group performance status of 0–1, adequate organ function, and no surgery within 2 weeks before enrollment. Patients without measurable lesions according to RECIST version 1.1 were eligible because the primary objective was estimation of baseline ctDNA *RAS*/*BRAF* discordance, which does not require measurable disease. Such patients were excluded from response, PFS, and OS analyses. Exclusion criteria were another malignancy within the previous 5 years, prior chemotherapy for colorectal cancer except adjuvant therapy completed at least 6 months earlier, acute or subacute intestinal obstruction, chronic inflammatory disease, or symptomatic peritoneal carcinomatosis. All participants provided written informed consent. The study was conducted in accordance with the Declaration of Helsinki, approved by local ethics committees (approval number 229023), prospectively registered with UMIN (UMIN000031177), and reported according to the STROBE statement. The full protocol has been published [21].

### 2.2. Tissue Genotyping

*RAS* and *BRAF* mutational analysis of tumor tissue was performed with the RASKET-B kit (MBL, Nagoya, Japan), a multiplex PCR-based assay that detects 48 hotspot mutations across *KRAS* (codons 12, 13, 59, 61, 117, and 146), *NRAS* (codons 12, 13, 59, 61, 117, and 146), and BRAF V600E [22]. All patients underwent tissue *RAS*/*BRAF* testing before study enrollment.

### 2.3. Plasma Collection and ddPCR Analysis

After obtaining written informed consent, 20 mL of peripheral blood was collected within 2 weeks before chemotherapy initiation. Cell-free DNA (cfDNA) was extracted from 1 mL of plasma and eluted in 30 µL with the Maxwell RSC cfDNA Plasma Kit (Promega, Madison, WI, USA). cfDNA concentration was quantified with the Qubit dsDNA HS Assay Kit (Thermo Fisher Scientific, Tokyo, Japan).

Baseline ctDNA was analyzed by ddPCR (QX200 Droplet Digital PCR System, Bio-Rad, Hercules, CA, USA) for *KRAS* (codons 12, 13, 61, and 146), *NRAS* (codons 12, 13, 61, and 146), *BRAF* V600E, and, in exploratory analyses, *PIK3CA*. The assays used manufacturer-provided PrimePCR mutation assays with TaqMan probe technology. A variant allele frequency (VAF) of ≥0.05% with ≥2 mutant droplets was considered positive. Each run included no-template controls, wild-type controls, and RNase P internal control assays.

For the primary analysis, discordance was defined as the absence of *RAS* and *BRAF* mutations in tumor tissue but the presence of a *KRAS*, *NRAS*, or *BRAF* mutation in ctDNA. *PIK3CA* was additionally assessed in an exploratory analysis to evaluate whether including this gene beyond *RAS* and *BRAF* would improve clinical discrimination.

### 2.4. Treatment and Outcome Assessment

Patients received FOLFOX, FOLFIRI, or FOLFOXIRI in combination with cetuximab or panitumumab according to physician choice. Tumor response was assessed by computed tomography using RECIST version 1.1. Conversion surgery was defined as surgical resection performed after systemic therapy with curative intent for lesions that had initially been considered unresectable or not indicated for upfront surgery. The decision to perform conversion surgery was made by the local multidisciplinary team at each participating institution, based on imaging findings after systemic therapy, technical feasibility of complete macroscopic resection, and the patient’s general condition. No prespecified central resectability criteria or central surgical review panel was used. Survival status was updated through October 2024.

### 2.5. End Points and Statistical Analysis

The primary endpoint was the baseline detection rate of ctDNA *RAS*/*BRAF* mutations among patients with tissue-confirmed *RAS*/*BRAF* wild-type tumors. Secondary endpoints were ORR, conversion surgery, PFS, and OS according to baseline concordance status. For *PIK3CA*, which was assessed as an exploratory endpoint, outcomes are presented descriptively given the limited number of mutation-positive cases identified.

PFS and OS were estimated by the Kaplan–Meier method and compared with the log-rank test. Multivariable Cox models included prespecified clinical covariates. Covariates were selected a priori based on established prognostic factors in mCRC; no additional variables were added post hoc, given the limited number of events relative to model parameters. Because conversion surgery occurs after treatment initiation, its effect on OS was evaluated by treating it as a time-dependent covariate to mitigate immortal-time bias, and the resulting models were treated as exploratory association analyses rather than causal models. Additional exploratory analyses included restricted mean survival time (RMST), time-dependent receiver operating characteristic (ROC) analysis for OS, and correlation between baseline ctDNA VAF and depth of response. All tests were two-sided, with *p* < 0.05 considered statistically significant.

The target sample size of approximately 100 patients was chosen to estimate the prevalence of baseline discordance with acceptable precision on the basis of our prior pilot study [15]. No formal hypothesis-driven power calculation was performed for survival comparisons.

## 3. Results

### 3.1. Patient Characteristics

**Figure 1:**
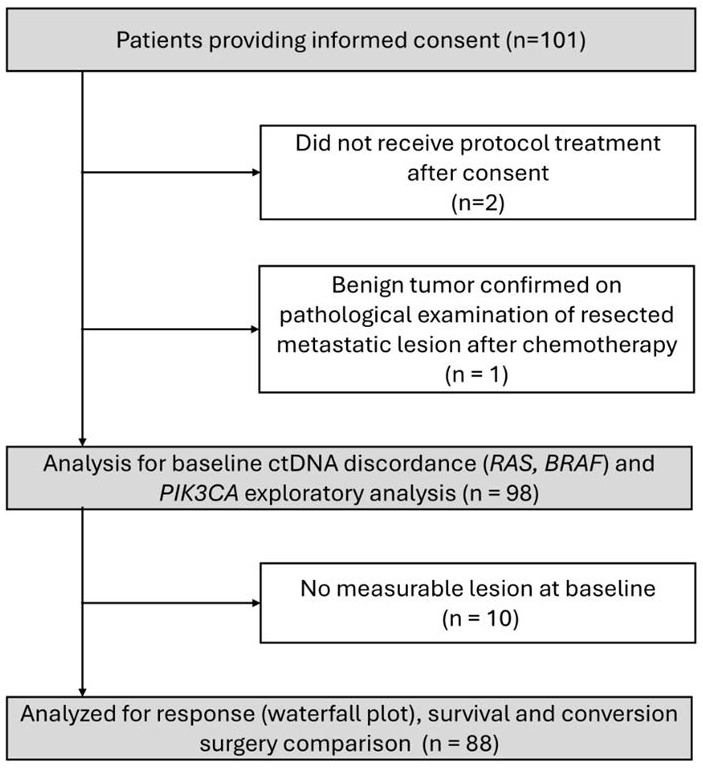
Patient selection flowchart.

A total of 101 patients were enrolled. One patient was excluded because the postoperative lesion was pathologically benign, and two patients consented but did not receive anti-EGFR-based chemotherapy. Consequently, 98 patients constituted the final analysis set. The patient selection flowchart is shown in Figure 1.

Baseline characteristics are summarized in Table 1. Most patients had performance status 0 (83.7%), left-sided primary tumors (94.9%), synchronous metastatic disease (76.5%), and liver metastases (69.4%). Baseline characteristics and treatment received according to disease measurability are summarized in Supplementary Table S1. Patients without measurable disease (n = 10) were more likely to have a performance status of 1 (50.0% vs. 12.5%) and less frequently had liver metastases (20.0% vs. 75.0%) compared with those with measurable disease (n = 88).

### 3.2. Baseline ctDNA Findings

ddPCR was successful in all pretreatment plasma samples (Table 2). Baseline ctDNA *RAS*/*BRAF* discordance was detected in 16 of 98 patients (16.3%). Detailed profiles of the 16 discordant cases are presented in Supplementary Table S2. When *PIK3CA* was included exploratorily, any mutation was identified in 20 of 98 patients (20.4%). Of the six *PIK3CA*-positive patients (6.1%; 95% CI, 2.8–12.7%), two also harbored *RAS* or *BRAF* mutations; the remaining four were *PIK3CA*-positive only. Owing to the small number of cases, no formal statistical comparison was performed; *PIK3CA*-specific outcomes are presented descriptively in Supplementary Table S3.

Among the 98 analyzed patients, no discordance was observed in the 10 patients without measurable target lesions. In the measurable-disease subgroup (n = 88), discordance was more frequent in patients with liver metastases (14/66, 21.2%) than in those without liver metastases (2/22, 9.1%), although this difference was not statistically significant. Notably, RAS mutations were detected only in patients with liver metastases. Liver-metastasis subgroup analyses are summarized in Supplementary Table S4.

### 3.3. Treatment Efficacy

**Figure 2:**
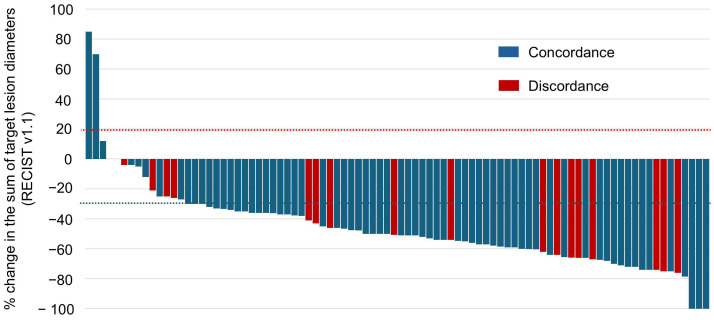
Waterfall plot of maximum tumor shrinkage. Each bar represents one patient with measurable disease (N = 88). The *y*-axis shows the best percentage change in the sum of target-lesion diameters from baseline according to RECIST version 1.1. The red line indicates a 20% increase corresponding to progressive disease, and the blue line indicates a 30% decrease corresponding to partial response.

Of the 98 enrolled patients, 88 (89.8%) had measurable disease per RECIST v1.1 and were eligible for ORR and DpR analyses; the remaining 10 patients with non-measurable target lesions only were excluded from these efficacy endpoints. Among the 88 patients with measurable disease, ORR was 84.1%, with complete response in 3 patients, partial response in 71, stable disease in 12, and progressive disease in 2. When *PIK3CA* was additionally excluded from the composite wild-type definition (“All wild,” N = 68), response rates and conversion surgery rates remained similar (Table 3 and Figure 2). Conversion surgery was performed in 44 patients (50.0%).

When stratified by baseline ctDNA–tissue concordance, ORR was similar in the concordant and discordant groups (84.7% vs. 81.3%). The conversion surgery rate was also comparable (48.6% vs. 56.3%). Median depth of response was 50% in the concordant group and 58% in the discordant group.

In an exploratory analysis, baseline ctDNA VAF was not significantly correlated with depth of response (Spearman’s ρ = 0.17, 95% CI −0.34 to 0.60, *p* = 0.54). The two patients with VAF ≥ 10% still achieved notable tumor shrinkage (Supplementary Figure S1).

### 3.4. Survival Outcomes

At the data cutoff, 47 deaths and 37 PFS events were recorded. In the OS analysis, 36 of 72 patients (50.0%) in the concordant group and 11 of 16 patients (68.8%) in the discordant group had died. In the PFS analysis, 30 of 72 patients (41.7%) in the concordant group and 7 of 16 patients (43.8%) in the discordant group had experienced disease progression; the remaining 44 patients who underwent conversion surgery were censored at the time of resection. The median follow-up calculated by the reverse Kaplan–Meier method was 47.3 months (range, 7.8–72.8).

**Figure 3:**
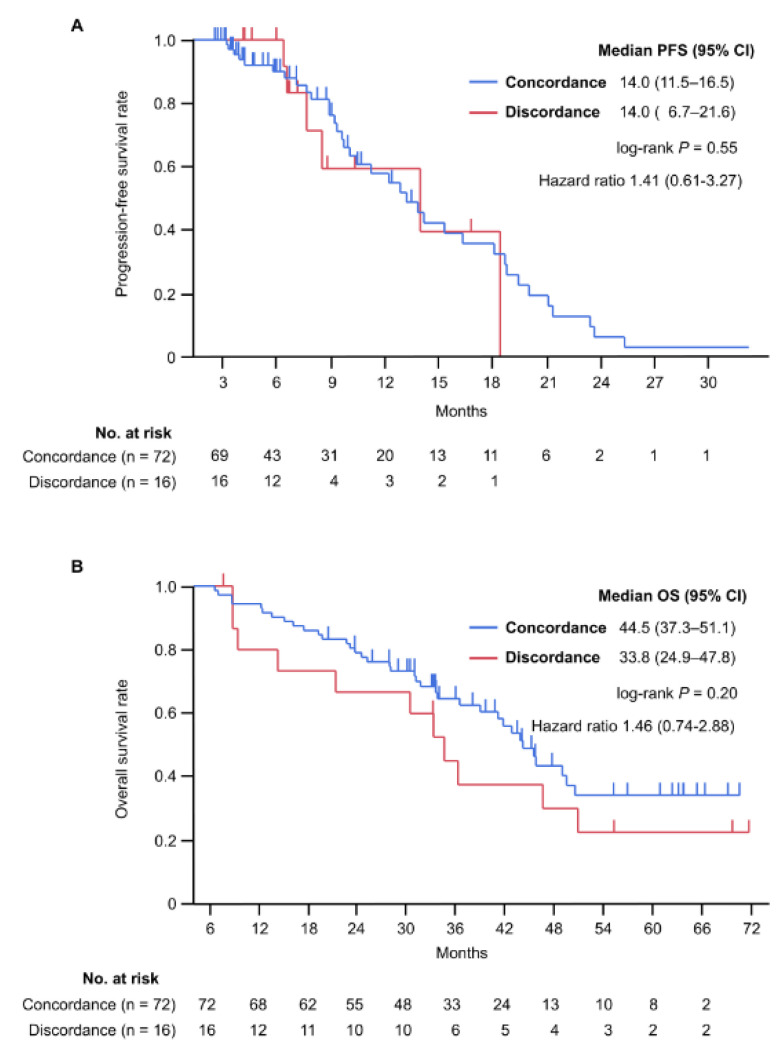
Kaplan–Meier curves for progression-free survival (**A**) and overall survival (**B**) according to baseline ctDNA–tissue concordance status. Median PFS was 14.0 months in both groups. Median OS was 44.5 months in the concordant group and 33.8 months in the discordant group.

Median PFS was 14.0 months (95% CI, 11.5–16.5) in the concordant group and 14.0 months (95% CI, 6.7–21.6) in the discordant group (Figure 3, log-rank *p* = 0.55). Median OS was 44.5 months (95% CI, 37.3–51.1) in the concordant group and 33.8 months (95% CI, 24.9–47.8) in the discordant group (log-rank *p* = 0.20). RMST and time-dependent ROC analyses are summarized in Supplementary Tables S5 and S6.

In the multivariable model for PFS, discordance was not independently associated with outcome (Table 4A). In the exploratory OS model that included post-baseline conversion surgery, discordance was not independently associated with OS, whereas conversion surgery was associated with improved OS (Table 4B).

## 4. Discussion

This prospective multicenter study demonstrates that restricted baseline ddPCR targeting only *RAS* and *BRAF* provides limited clinical discrimination for excluding patients with tissue-confirmed *RAS*/*BRAF* wild-type mCRC from first-line anti-EGFR-based combination therapy. Although 16.3% of patients exhibited baseline plasma–tissue discordance, these discordant cases still achieved remarkably high objective response rates (81.3%) and robust conversion surgery rates (56.3%). Furthermore, no statistically significant difference was observed in PFS or OS between the concordant and discordant groups.

Our results do not contradict the value of comprehensive genomic profiling for ctDNA-based negative hyperselection; rather, they highlight that restricted hotspot ddPCR is insufficient as a stand-alone tool to exclude patients from intensive first-line therapy, particularly when conversion surgery is the therapeutic goal. In the PARADIGM biomarker analysis, comprehensive baseline ctDNA profiling using a multigene NGS panel successfully identified subgroups with markedly different outcomes following first-line panitumumab-based therapy [20]. Similarly, recent real-world data and large-scale pooled analyses have advocated for stringent patient selection to optimize EGFR antibody therapies [23,24,25].

Several biological and technical factors likely explain why minor discordant clones detected by ddPCR did not dictate primary resistance in our cohort. First, most discordant plasma mutations were detected at very low variant allele frequencies (VAFs). Recent translational studies, such as the Valentino study, have shown that baseline ctDNA VAF strongly correlates with overall tumor burden and can stratify patient outcomes [26,27]. A higher ctDNA fraction is generally associated with decreased survival across various targeted therapies [27]. In our study, the low VAFs in the discordant group suggest the presence of minor, subclonal populations rather than dominant resistant drivers. In the first-line setting, the vast majority of the tumor bulk remains highly sensitive to the combination of cytotoxic doublets and EGFR blockade. This susceptibility enables substantial and rapid tumor shrinkage (median DpR of 58% in the discordant group), effectively overriding the theoretical resistance of the minor clones.

Second, ctDNA shedding dynamics are heavily influenced by the anatomical distribution of metastases. In our cohort, *RAS* mutations were detected exclusively in patients with liver metastases, and no discordance was observed in patients lacking measurable target lesions. The complete absence of discordance in this subgroup is consistent with the hypothesis that a minimum tumor burden is required to shed detectable mutant ctDNA fragments above the ddPCR positivity threshold; in patients with only non-measurable disease, the circulating tumor DNA fraction may fall below the level of reliable detection regardless of underlying clonal heterogeneity. This pattern aligns closely with previous reports indicating that liver metastases shed significantly more ctDNA into the systemic circulation compared to lung-only, lymph node, or peritoneal-dominant disease [28]. In patients with low-shedding metastatic profiles, a negative result on a restricted ddPCR assay must be interpreted with extreme caution, as it may represent a false-negative liquid biopsy rather than true biological wild-type status. Consequently, liquid biopsy should complement, not overrule, comprehensive tissue genotyping in these specific clinical scenarios.

Third, the mutational coverage between the standard tissue assay and our plasma assay was not perfectly identical. The RASKET-B tissue panel covers codons 59 and 117 in *KRAS* and *NRAS* [22], whereas our plasma ddPCR panel was limited to codons 12, 13, 61, and 146, alongside *BRAF* V600E. Therefore, some rare alterations detectable in tissue could not be directly interrogated in plasma, meaning the observed discordance rate (16.3%) may slightly underestimate the full extent of molecular heterogeneity. Additionally, when *PIK3CA* was exploratorily added to our composite index, the discriminative ability (AUC) for survival outcomes was paradoxically reduced. This reduction likely occurs because *PIK3CA* mutations are generally considered less specific for intrinsic anti-EGFR resistance compared to *RAS* or *BRAF* mutations, and their inclusion may dilute the predictive power of the panel by reflecting broad subclonal diversity rather than targeted treatment failure.

A critical and highly practical finding of our study is the profound impact of conversion surgery. Multivariable analysis identified conversion surgery as the strongest independent predictor of improved overall survival (HR 0.397, *p* = 0.02). The high conversion rate achieved even in the discordant group (56.3%) strongly suggests that achieving surgical resection of the remaining macroscopic disease was associated with markedly improved prognosis. These findings echo recent evaluations emphasizing that while baseline liquid biopsies, such as those analyzed in the FIRE-4 and FIRE-4.5 studies, provide significant prognostic information regarding intrinsic biology [29,30], they should not universally deter aggressive multidisciplinary surgical interventions. From a pragmatic clinical perspective, low-level baseline plasma positivity on a restricted ddPCR panel should be interpreted as a trigger for more comprehensive molecular contextualization rather than as an automatic exclusion criterion. When conversion to resection is the treatment objective, decision making should integrate tissue genotyping, metastatic distribution, ctDNA fraction or VAF, radiologic response kinetics, and repeated multidisciplinary review of resectability. Broader panel-based ctDNA sequencing may be most useful when plasma–tissue discordance is detected or when the clinical course appears biologically inconsistent with the known tissue profile.

This study has several important design limitations. The cohort was inherently enriched for patients selected by treating physicians for intensive first-line therapy and potential local treatment, introducing a selection bias toward better overall outcomes than typically observed in unselected stage IV populations. The relatively small sample size—particularly the limited number of discordant cases (N = 16)—restricts statistical power, increasing the risk of a Type II error in the survival analyses. Notably, the observed 11-month numerical difference in median OS (44.5 vs. 33.8 months) and the higher event rate in the discordant group (68.8% vs. 50.0%) may represent a clinically relevant trend; however, these observations should be regarded as hypothesis-generating. The limited number of events (47 deaths; 37 PFS events) constrained the number of covariates that could be reliably included in multivariable models. Variables such as chemotherapy backbone, anti-EGFR antibody type, liver-only disease, and baseline CEA could not be formally evaluated as covariates without risking model overfitting; their potential confounding effects remain unquantified. Because resectability and the indication for conversion surgery were determined by local multidisciplinary teams without central surgical review, interinstitutional variability in surgical decision-making may have occurred. In addition, pathological resection margin status was not systematically recorded. Although all conversion surgeries were recorded as macroscopically complete resections with no gross residual disease, the proportion of patients achieving a microscopically negative margin could not be confirmed. These limitations preclude precise characterization of surgical quality and may affect the interpretation of conversion surgery as a prognostic variable. Finally, our study utilized only baseline ctDNA assessment. Longitudinal ctDNA profiling during anti-EGFR therapy may help distinguish minor baseline subclones—which may be suppressed by cytotoxic combination chemotherapy—from de novo resistant clones emerging under selective pressure, thereby providing actionable information to guide treatment modification, maintenance strategies, or rechallenge decisions. Whether broader NGS-based ctDNA profiling could more precisely identify the subset of discordant patients who would ultimately fail to benefit from anti-EGFR-based induction therapy warrants further investigation.

## 5. Conclusions

Restricted baseline hotspot ctDNA testing for *KRAS*, *NRAS*, and *BRAF* by ddPCR identified plasma RAS/BRAF discordance in a subset of patients with tissue-confirmed *RAS*/*BRAF* wild-type mCRC, but it did not reliably predict failure to achieve objective response or conversion surgery. When rapid cytoreduction for curative-intent resection is the treatment goal, limited *KRAS*/*NRAS*/*BRAF*-targeted ctDNA testing alone should not justify withholding first-line anti-EGFR-based combination therapy; however, given the small number of discordant cases, a clinically meaningful effect of baseline ctDNA discordance cannot be definitively excluded. Future biomarker strategies should integrate broader NGS panels, longitudinal sampling, and MDT-driven surgical evaluation.

## Figures and Tables

**Table 1:** Baseline patient characteristics (N = 98).

Characteristic	Value
Age, median (range)	67 (38–79)
Male	68 (69.4%)
Performance status	0:82 (83.7%); 1:16 (16.3%)
Primary tumor location	Right-sided: 5 (5.1%); Left-sided: 93 (94.9%)
Timing of metastasis	Synchronous: 75 (76.5%); Metachronous: 23 (23.5%)
Metastatic sites	Liver: 68 (69.4%); Lung: 29 (29.6%); Peritoneum: 13 (13.3%); Lymph node: 25 (25.5%); Ovary: 5 (5.1%); Local recurrence: 18 (18.4%)
Anti-EGFR antibody	Cetuximab: 37 (37.8%); Panitumumab: 61 (62.2%)

**Table 2:** Baseline ctDNA mutation detection by ddPCR (N = 98).

Category	Patients with Mutation	95% CI
Any mutation, including *PIK3CA*	20 (20.4%)	13.6–29.4
*RAS* mutation	12 (12.2%)	7.1–20.2
*KRAS* mutation	8 (8.2%)	4.2–15.3
*NRAS* mutation	4 (4.1%)	1.6–10.0
*BRAF* mutation	4 (4.1%)	1.6–10.0
*PIK3CA* mutation	6 (6.1%)	2.8–12.7
Multiple mutations	2 (2.0%)	0.6–7.1

Primary endpoint: detection rate of ctDNA *RAS*/*BRAF* mutations. Exploratory analyses additionally included *PIK3CA*. The discordance rate (tissue wild-type, ctDNA *RAS*/*BRAF*-positive) was 16/98 (16.3%; 95% CI, 10.3–24.9%).

**Table 3:** Efficacy of first-line anti-EGFR-based chemotherapy in patients with measurable disease (N = 88).

Outcome	All Patients (N = 88)	Concordance (N = 72)	Discordance (N = 16)	All Wild (N = 68)
ORR	74 (84.1%)	61 (84.7%)	13 (81.3%)	58 (85.3%)
CR	3 (3.4%)	3 (4.2%)	0 (0.0%)	3 (4.4%)
PR	71 (80.7%)	58 (80.6%)	13 (81.3%)	55 (80.9%)
SD	12 (13.6%)	9 (12.5%)	3 (18.8%)	8 (11.8%)
PD	2 (2.3%)	2 (2.8%)	0 (0.0%)	2 (2.9%)
Depth of response	51%	50%	58%	50%
Conversion surgery	44 (50.0%)	35 (48.6%)	9 (56.3%)	34 (50.0%)

Abbreviations: ORR, overall response rate; CR, complete response; PR, partial response; SD, stable disease; PD, progressive disease. All wild was defined as the absence of *KRAS*, *NRAS*, *BRAF*, and *PIK3CA* mutations in ctDNA.

**Table 4:** (**A**). Multivariable analysis of progression-free survival. (**B**). Exploratory multivariable analysis of overall survival (incorporating conversion surgery as a time-dependent covariate).

(**A**)			
Variable	HR	95% CI	*p* value
Age	1.032	0.994–1.071	0.10
Liver metastasis (yes vs. no)	1.858	0.829–4.161	0.13
Multiple organ metastases (yes vs. no)	0.626	0.293–1.336	0.23
Performance status (1 vs. 0)	1.271	0.443–3.642	0.66
Left-sided tumor (yes vs. no)	0.417	0.050–3.498	0.42
Discordance (yes vs. no)	0.784	0.233–2.655	0.70
(**B**)			
Variable	HR	95% CI	*p* value
Age	1.002	0.964–1.041	0.91
Liver metastasis (yes vs. no)	1.374	0.628–3.005	0.43
Multiple organ metastases (yes vs. no)	1.120	0.538–2.334	0.76
Performance status (1 vs. 0)	1.732	0.453–6.622	0.42
Left-sided tumor (yes vs. no)	0.090	0.010–0.800	0.03
Discordance (yes vs. no)	0.683	0.266–1.754	0.43
Conversion surgery (yes vs. no)	0.397	0.185–0.851	0.02

(**A**) Analysis restricted to patients with measurable disease (N = 88). (**B**) Analysis conducted in the survival analysis set (N = 88). This model included conversion surgery as a post-baseline variable and should be interpreted as exploratory.

## Data Availability

The datasets generated and/or analyzed during the current study are available from the corresponding author upon reasonable request.

## Supplementary Materials

The following supporting information can be downloaded at: https://www.mdpi.com/article/10.3390/cancers18111688/s1, Figure S1: Variant allele frequency (VAF) versus depth of response (DpR); Table S1: Baseline characteristics and treatment received in patients with measurable versus non-measurable disease; Table S2: Discordant cases; Table S3: Characteristics and outcomes of PIK3CA-positive cases in the exploratory analysis; Table S4: Liver-metastasis subgroup analysis in patients with measurable disease; Table S5: Restricted mean survival time (RMST); Table S6: Time-dependent ROC analysis for overall survival (OS).
