# Supplementary material for: Clinical Impact of Baseline ctDNA RAS/BRAF Mutations on Conversion Surgery and Outcomes in First-Line Anti-EGFR Therapy for Advanced Colorectal Cancer
Source: Cancers (Basel). 2026 May 22;18(11):1688. doi: 10.3390/cancers18111688 (PMC13255658; doi:10.3390/cancers18111688)
Supplement: Supplementary file 1 [file cancers-18-01688-s001.zip › cancers-4271314-supplementary.pdf]

cancers

MDPI

Supplementary Files

Clinical Impact of Baseline ctDNA RAS/BRAF Mutations on Conversion Surgery and Outcomes in First-Line Anti-EGFR Therapy for Advanced Colorectal Cancer

Takeshi Yamada, Takeshi Nagasaka, Nobuhisa Matsuhashi, Takao Takahashi, Keiji Hirata, Yuki Nakamura, Kiichi Sugimoto, Keiji Koda, Kazuhiro Hiramatsu, Hiroshi Matsuoka, Hidekazu Kuramochi, Akihisa Matsuda, Hideyuki Ishida, Kozo Kataoka, Hajime Yokomizo, Yoshinori Kagawa, Mitsukuni Suenaga and Hiroshi Yoshida

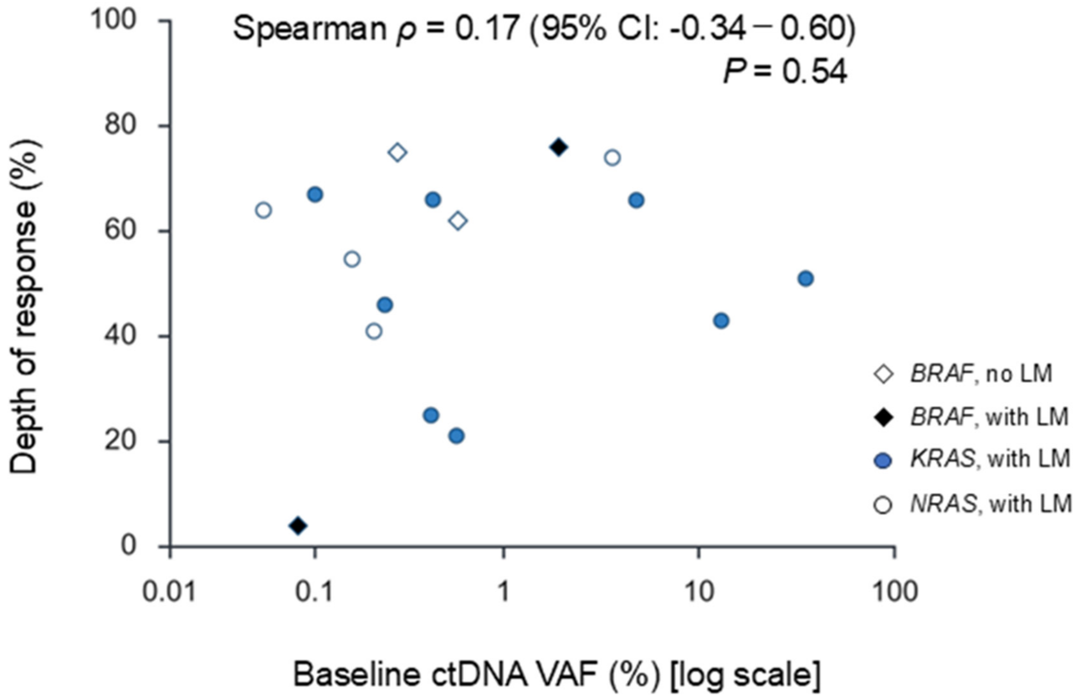

Figure S1. Variant allele frequency (VAF) versus depth of response (DpR). Scatterplot of baseline ctDNA VAF (log scale) versus DpR, defined as the maximum percentage change in the sum of target-lesion diameters per RECIST version 1.1. The overall association between VAF and DpR was not significant (Spearman's ϱ = 0.17, 95% CI −0.34 to 0.60, P = 0.54). Two KRAS-mutant cases with VAF ≥10% (35.2% and 13.0%) still achieved substantial shrinkage.

 https://doi.org/10.3390/cancers18111688

Table S1. Baseline characteristics and treatment received in patients with measurable versus non-measurable disease.

Characteristic	With measurable disease (n=88)	Without measurable disease (n=10)
Age, median (range)	67 (41–79)	69 (38–79)
Male	62 (70.5%)	6 (60.0%)
Performance status	0: 77 (87.5%); 1: 11 (12.5%)	0: 5 (50.0%); 1: 5 (50.0%)
Primary tumor location	Right-sided: 5 (5.7%)<br>Left-sided: 83 (94.3%)	Right-sided: 0 (0.0%)<br>Left-sided: 10 (100.0%)
Timing of metastasis	Synchronous: 69 (78.4%)<br>Metachronous: 19 (21.6%)	Synchronous: 6 (60.0%)<br>Metachronous: 4 (40.0%)
Metastatic sites	Liver: 66 (75.0%); Lung: 26 (29.5%); Peritoneum: 10 (11.4%); Lymph node: 21 (23.9%); Ovary: 5 (5.7%); Local recurrence: 15 (17.0%)	Liver: 2 (20.0%); Lung: 3 (30.0%); Peritoneum: 3 (30.0%); Lymph node: 4 (40.0%); Ovary: 0 (0.0%); Local recurrence: 3 (30.0%)
Anti-EGFR antibody	Cetuximab: 35 (39.8%)<br>Panitumumab: 53 (60.2%)	Cetuximab: 2 (20.0%)<br>Panitumumab: 8 (80.0%)
Conversion surgery	44 (50.0%)	0 (0.0%)

https://doi.org/10.3390/cancers18111688

Table S2. Discordant cases.

Case	Age	Sex	Tumor location	Synchro-nous	Liver me-tastasis	VAF	PFS (months)	OS (months)
1	45	F	S	Yes	Yes	0.28 (B)	8.9	51.7
2	58	M	R	Yes	Yes	35.21 (K)	6.5	21.7
3	65	M	R	Yes	No	1.92 (B)	8.6	47.3
4	60	M	R	Yes	Yes	0.16 (N)	10.5	30.9
5	60	F	R	Yes	Yes	0.57 (K)	4.3	9.0
6	75	M	R	Yes	No	0.08 (B)	4.2	70.8 (alive)
7	70	M	R	Yes	Yes	0.10 (K)	6.1	72.8 (alive)
8	55	M	S	No	Yes	4.80 (K)	14.2	33.8
9	71	M	S	Yes	Yes	0.24 (K)	6.8	35.1
10	50	F	S	Yes	Yes	3.62 (N)	6.8	36.8
11	40	F	R	Yes	Yes	0.43 (K)	17.0	56.1 (alive)
12	62	M	S	Yes	Yes	13.04 (K)	4.7	14.5
13	74	M	S	No	Yes	0.21 (N)	7.8	7.8
14	70	M	S	Yes	Yes	0.05 (N)	18.7	33.7 (alive)
15	72	M	S	Yes	Yes	0.42 (K)	7.2	9.0
16	70	M	S	Yes	Yes	0.58 (B)	6.7	9.6

Abbreviations: F, female; M, male; S, sigmoid colon; R, rectum; VAF, variant allele frequency; K, *KRAS*; N, *NRAS*; B, *BRAF*.

https://doi.org/10.3390/cancers18111688

Table S3. Characteristics and outcomes of *PIK3CA*-positive cases in the exploratory analysis.

Case	Age	Sex	Tumor location	Synchronous	Liver metastasis	VAF	PFS (months)	OS (months)
10	50	F	S	Yes	Yes	3.62 (N)<br>0.40 (P)	6.8	36.8
16	70	M	S	Yes	Yes	0.58 (B)<br>0.82 (P)	6.7	9.6
17	75	F	R	Yes	Yes	0.05 (P)	7.2	8.8
18	64	M	S	No	No	0.12 (P)	25.7	44.1
19	46	M	S	No	Yes	18.23 (P)	13.0	24.1
20	69	M	D	Yes	Yes	39.93 (P)	4.1	34.0

Abbreviations: F, female; M, male; S, sigmoid colon; R, rectum; VAF, variant allele frequency; K, *KRAS*; N, *NRAS*; B, *BRAF*; P, *PIK3CA*.

Table S4. Liver-metastasis subgroup analysis in patients with measurable disease (N = 88).

Variable	With liver metastasis (N = 66)	Without liver metastasis (N = 22)	P value
Discordance	14/66 (21.2%)	2/22 (9.1%)	0.34
RAS mutation	12/66 (18.2%)	0/22 (0.0%)	0.03
KRAS mutation	8/66 (12.1%)	0/22 (0.0%)	—
NRAS mutation	4/66 (6.1%)	0/22 (0.0%)	—
BRAF mutation	2/66 (3.0%)	2/22 (9.1%)	0.10
PIK3CA mutation	5/66 (7.6%)	1/22 (4.5%)	1.00
Median PFS (95% CI)	13.0 months (8.8–17.3)	18.3 months (9.6–27.0)	Log-rank 0.07; Wilcoxon 0.16
Median OS (95% CI)	39.6 months (30.5–48.6)	46.5 months (41.5–51.1)	Log-rank 0.31; Wilcoxon 0.19

Discordance was defined as tissue RAS/BRAF wild-type with ctDNA RAS/BRAF positivity.

Table S5. Restricted mean survival time (RMST).

Endpoint	Time horizon	Discordant (months)	Concordant (months)	Difference	95% CI	P value
PFS	1 year	8.8	8.8	0.0	−1.8 to 1.7	0.97
PFS	2 years	9.3	8.8	0.5	−1.4 to 2.3	0.62
PFS	3 years	9.3	8.8	0.5	−1.4 to 2.3	0.62
OS	1 year	10.5	9.4	1.1	−0.8 to 3.0	0.26
OS	2 years	19.3	17.0	2.3	−1.5 to 6.1	0.23
OS	3 years	26.4	23.2	3.2	−1.4 to 7.8	0.17

Abbreviations: PFS, progression-free survival; OS, overall survival; RMST, restricted mean survival time.

https://doi.org/10.3390/cancers18111688

Table S6. Time-dependent ROC analysis for OS.

Time horizon	ctDNA *RAS/BRAF* positive (AUC, 95% CI)	ctDNA *RAS* positive only (AUC, 95% CI)	Any mutation including *PIK3CA* (AUC, 95% CI)
1 year	0.740 (0.589–0.871)	0.512 (0.385–0.639)	0.456 (0.156–0.756)
2 years	0.668 (0.504–0.808)	0.544 (0.417–0.671)	0.387 (0.087–0.687)
3 years	0.778 (0.624–0.893)	0.651 (0.524–0.778)	0.421 (0.121–0.721)
4 years	0.709 (0.568–0.844)	0.693 (0.566–0.820)	0.398 (0.098–0.698)

The index test was coded as binary at baseline. AUCs were estimated by the timeROC method.

https://doi.org/10.3390/cancers18111688
